# Gastric adenocarcinoma burden and late‐stage diagnosis in Latino and non‐Latino populations in the United States and Texas, during 2004–2016: A multilevel analysis

**DOI:** 10.1002/cam4.4175

**Published:** 2021-08-19

**Authors:** Dorothy Long Parma, Susanne Schmidt, Edgar Muñoz, Amelie G. Ramirez

**Affiliations:** ^1^ Department of Population Health Sciences School of Medicine The University of Texas Health Science Center at San Antonio San Antonio Texas USA; ^2^ Mays Cancer Center The University of Texas Health Science Center at San Antonio San Antonio Texas USA

**Keywords:** gastric adenocarcinoma, health disparities, late‐stage disease, Latinos, multilevel analysis

## Abstract

**Background:**

Gastric cancer disproportionately affects Latinos, but little is known about regional effects and risk factors. We compared primary incidence, late‐stage diagnosis, and risk factors for gastric adenocarcinoma (GCA) from 2004 to 2016 in Latinos and non‐Latinos in the United States, Texas (TX), and South Texas (STX).

**Methods:**

We collected case data from Surveillance, Epidemiology, and End Results (SEER) and the Texas Cancer Registry. We generated average annual age‐adjusted incidence rates, rate ratios (RRs), and 95% confidence intervals (CIs) using SEER*Stat software and analyzed the cases by anatomic site, demographics, and county‐level risk factors using SAS 9.4. We constructed multilevel logistic regression models for late‐stage GCA, adjusting for patient‐ and county‐level characteristics.

**Results:**

Latinos had higher overall GCA incidence rates in all regions, with the greatest disparities in overlap GCA in STX males (RR 4.39; 95% CI: 2.85, 6.93). There were no differences in cardia GCA rates for non‐Hispanic Whites (NHWs) and Latino women in all regions. Younger patients, patients with overlapping or not otherwise specified (NOS) lesions, and patients diagnosed during 2012–2016 had higher odds of late‐stage GCA. The stratification by location showed no differences in late‐stage disease between NHWs and Latinos. The stratification by anatomic site showed Latinos with cardia GCA were more likely to have late‐stage GCA than NHWs (OR: 1.13, *p* = 0.008). At the county level, higher odds of late‐stage GCA were associated with medium and high social deprivation levels in TX without STX (OR: 1.25 and 1.20, *p* = 0.007 and 0.028, respectively), and medium social deprivation index (SDI) in patients with NOS GCA (OR: 1.21, *p* = 0.01).

**Conclusions:**

STX Latinos experience greater GCA disparities than those in TX and the United States. Younger age and social deprivation increase the risk for late‐stage GCA, while Latinos and women are at higher risk specifically for late‐stage cardia GCA. There is a need for population‐specific, culturally responsive intervention and prevention measures, and additional research to elucidate contributing risk factors.

## Abstract 2

Latinos had higher gastric adenocarcinoma incidence than non‐Latinos in the U.S., Texas and South Texas from 2004 to 2016. Younger age and social deprivation increased risk of late‐stage diagnosis, while Latino ethnicity and female gender increased risk of late‐stage gastric adenocarcinoma of the cardia specifically.

## 1 INTRODUCTION

Cancer is the leading cause of death among Latinos, surpassing heart disease, the primary cause of mortality among non‐Hispanic Whites (NHWs).^1^, ^2^ Latinos comprise 18.5% of the US population^3^ and are projected to reach 28.6% by 2060.^4^ Latinos comprise 39.7% of all Texans, and Texas Latinos account for about 17% of the entire US Latino population. South Texas (STX), a 38‐county region encompassing San Antonio south to the Lower Rio Grande Valley along the Texas–Mexico border, is nearly 70% Latino.^5^

Compared to NHWs, Latinos have higher risk of developing multiple cancers, including gastric cancer (GC).^6^ GC is the third leading cause of cancer death worldwide^7^ and is the sixth‐ and eighth leading cause for Texas men and women, respectively, in 2012–2016.^1^ Mortality rates from 2012 to 2016 were twice as high for Latino than NHW men and 2.4 times higher for Latino than NHW women. Gastric adenocarcinomas (GCAs) account for approximately 90% of all GCs and are the focus of this study.

GC incidence has been increasing in persons aged <50 years and Latino women.^7^, ^8^ In 2018, the probability of developing invasive GC at any age was twice as high in Latino versus NHW men (1.6 vs. 0.8), and three times higher in Latino versus NHW women (1.2 vs. 0.4).^1^ Moreover, Latinos are diagnosed at younger ages and more advanced stages compared to other ethnic groups.^1^, ^9^, ^10^

Latinos are disproportionately vulnerable to cancer due to increased poverty, decreased education, low or no insurance, and high prevalence of risk factors like obesity^1^ and heavy alcohol consumption.^6^ About 22% of STX residents on average had an income below 150% of the federal poverty line in 2018 (range 7.3%–35%), compared with the US national average of 12%.^11^ Our previously published review of health conditions in STX versus Texas and the US examined data on health insurance, obesity, alcohol use, and smoking.^12^ For the period 2007–2010, an estimated 30% of STX residents were uninsured compared to 23% in the rest of Texas and 15% nationwide. Latinos had the highest uninsured rate (40%). STX also had the highest percentage of obese adults (32.7% vs. 29.1% in the rest of Texas and 27% in the United States). STX Latinos were more obese (37.9%) than NHWs (24.6%). STX diabetes rates were likewise higher than the rest of Texas and national estimates (11.6% vs. 9.3% and 8.9%, respectively), with Latinos again accounting for larger proportions of diabetics. Although alcohol use and smoking were similar in all three regions, binge drinking rates were higher in STX (17.4%) than the rest of Texas (14.5%) and the nation (15.1%).

The social deprivation index (SDI) is a composite measure of county‐level deprivation based on seven American Community Survey (ACS) measures for income, education, employment, housing, household characteristics, and transportation.^13^ The SDI has been previously used to quantify the socioeconomic variation in health outcomes, including diabetes prevalence, odds of received colon and cervical cancer screening, and surgical treatment of head and neck cancers.^14^, ^15^

Previous studies have reported mixed results on ethnic disparities in GCA trends. Two studies using Surveillance, Epidemiology, and End Results (SEER) and National Program of Cancer Registries data from 1992 to 1998 and 1976 to 2007 found that Caucasians had a 1.7 times higher GCA incidence rate and increased gastric body GCs, respectively, compared to White Latinos.^16^, ^17^ In contrast, studies in Southern California and Texas (using Texas Cancer Registry data) found increased odds of GC in racial/ethnic minorities and a higher GCA incidence in Latinos, respectively (with a 29% excess risk in El Paso County), compared to the NHW population.^18^, ^19^ Short time frame, lack of GCA anatomic site data, and failure to compare Texas data with national trends were limitations of these studies. Finally, Balakrishnan and colleagues’ retrospective cohort study of 299 non‐cardia GC cases between 2005 and 2015 in the Harris County, Texas public medical system found increasing incidence among Hispanics/Latinos but not NHWs or Blacks.^20^

The current study aimed to compare the rates and late‐stage diagnosis for all primary GCA in Latinos and NHWs from 2004 to 2016 in the United States, Texas, and STX using multilevel modeling. The goal was to elucidate existing differences and the influence of specific risk factors in order to facilitate novel interventions aimed at reducing gastric adenocarcinoma incidence in specific high‐risk, diverse populations.

## 2 METHODS

### 2.1 Ethical statement

The study was exempted from review by the Institutional Review Board at UT Health Science Center at San Antonio, and conforms to the Declaration of Helsinki and the US Federal Policy for the Protection of Human Subjects.

### 2.2 Data

#### 2.2.1 Gastric cancer data

We obtained de‐identified data via Limited‐Use Data Agreements from the US SEER Program Registries^21^ and the Texas Cancer Registry (TCR) at the Texas Department of State Health Services.^22^

SEER is a population‐based cancer registry system that has collected individual‐level data provided by participating registries in select states since 1971. This study used the SEER 21‐registry grouping excluding Alaska due to lack of county‐level data.

While not included in SEER, the TCR is an identically organized population‐based registry of all 254 Texas counties following all SEER standards and coding criteria. The registry has earned the North American Association of Central Cancer Registries (NAACCR) Gold Certification for data quality and completeness.^23^

#### 2.2.2 Inclusion and exclusion

We coded primary GCAs according to the International Classification of Diseases for Oncology (ICD‐O) GCA morphology codes (81403, 84903, 81453, 81443, 82113, 82102, 84803, 80103, and 85603).^24^ We subdivided all cases by anatomic location according to the ICD 10th Edition Clinical Modification into cardia/gastroesophageal junction (C16.0), non‐cardia (C16.1–C16.6), overlapping lesion (C16.8), and not otherwise specified (NOS, C16.9).^25^ The coding followed the example of prior studies.^20^ We limited our analysis to patients aged 20+ diagnosed in 2004–2016, for whom GCA was their first cancer. We further excluded cases without valid county codes, cases reported based on an autopsy/death certificate, those with unknown stage, and those who had missing county‐level data. The resulting datasets included 142,068 GCA cases for rate analyses and 75,761 GCA cases for late‐stage diagnosis.

#### 2.2.3 County‐level data

We used county‐level data from the 2018 County Health Rankings (CHR) for health behaviors and risk factors.^26^ CHR combines several data sources, including the American Community Survey (ACS), the Behavioral Risk Factor Surveillance System, and the USDA Food Environment Atlas. We added the 2015 SDI to estimate the county‐level deprivation and act as a proxy for SES.

### 2.3 Measures

#### 2.3.1 Rates

We obtained population denominators used for all rate calculations from SEER.^21^ We defined ethnicity using the NAACCR Hispanic/Latino Identification Algorithm, version 2.2.1.^27^ We selected GCA incident cases from 2004 to 2016 from the 21 SEER registries (cumulative population at risk = 194 million Latino person‐years, 655 million NHW person‐years); and TCR for all of Texas (76 million Latino, 116 million NHW person‐years) and the 38‐county STX region (25 million Latino, 12 million NHW person‐years). We generated average annual age‐specific, age‐adjusted GCA incidence rates, rate ratios (RRs), and their corresponding 95% confidence intervals (CIs) for NHWs, Non‐Hispanic Blacks, Latinos, and Non‐Hispanic Others in the SEER and TCR datasets using SEER*Stat software v8.3.8 (2020, National Cancer Institute, Bethesda, MD). We used standard age groups for rates estimation.

#### 2.3.2 Logistic regression analysis outcome

We defined the late‐stage GCA diagnosis as known metastatic disease (i.e., distant site(s)/node(s) involved) using the combined Summary Stage 2000 variable in the SEER and TCR data versus non‐late‐stage (including localized and regional but not unknown).

#### 2.3.3 Patient characteristics

We included age at diagnosis (20–39, 40–64, vs. 65+), sex (male vs. female), race/ethnicity (Non‐Hispanic Black, Latino, Non‐Hispanic Other, vs. NHW), site (non‐cardia, overlap, NOS vs. cardia), year of diagnosis (2008–2011, 2012–2016, vs. 2004–2007), and location (Texas without South Texas [TX], the 38‐county STX, vs. SEER). Categories for year of diagnosis were chosen to achieve as equal a distribution of patients over time as possible. We also included reporting source as previous research has shown this may impact the unknown stage.^28^

#### 2.3.4 County‐level predictors

From the CHR, we included percent current smokers, percent obese, percent reporting excessive alcohol consumption (i.e., binge/heavy drinking), and the food environment index, an indicator of environmental access to healthy food options. All CHR measures were z‐scored prior to analysis. We included the SDI (21–79, 80–100 [most deprived], vs. 0–20 [least deprived]) as a measure of socioeconomic deprivation.

### 2.4 Analytical approach

We report the descriptive univariate and bivariate statistics for late‐stage GCA diagnosis, patient characteristics, and county‐level predictors. To examine the effect of patient‐ and county‐level predictors on the diagnosis of late‐stage GCA, we constructed multilevel logistic regression models adjusting for county‐level clustering. We used a nested approach, first only including location (i.e., SEER, TX, STX, Model 1; Table 1) and reporting source, then adding patient‐level predictors (Model 2), followed by county‐level predictors (Model 3). We stratified the final model by location (SEER, TX, STX; Table 2) and anatomic site (cardia, non‐cardia, overlapping, and NOS; Table 3).

**TABLE 1:** Descriptive statistics and multilevel logistic regression models for late‐stage GCA diagnosis, adults 20+, 2004–2016

*n*	Descriptive statistics			Logistic regression models					
	All GCA patients	Patients with late‐stage GCA DX		Model 1		Model 2		Model 3	
	75,761	33,071		75,761		75,761		75,761	
	% (*n*)	% (*n*)	*p* value	OR	*p* value	OR	*p* value	OR	*p* value
Late‐stage GCA DX			—						
Yes	43.65% (33,071)	—		—	—	—	—	—	—
No	56.35% (42,690)	—		—	—	—	—	—	—
Location			<0.0001						
SEER	85.48% (64,758)	43.50% (28,170)		Ref		Ref		Ref	
TX (w/o) STX	11.43% (8663)	45.71% (3960)		1.043	0.1815	0.940	0.0650	**0.917**	**0.0167**
STX	3.09% (2340)	40.21% (941)		0.901	0.0656	**0.814**	**0.0011**	**0.810**	**0.0007**
Sex			0.3020						
Female	36.39% (27570)	43.41% (11967)				Ref		Ref	
Male	63.61% (48191)	43.79% (21104)		—	—	1.017	0.3025	1.017	0.2809
Age at DX			<0.0001						
20–39	4.02% (3047)	61.67% (1879)		—	—	**2.462**	**<0.0001**	**2.463**	**<0.0001**
40–64	40.37% (30,584)	48.83% (30,584)		—	—	**1.511**	**<0.0001**	**1.511**	**<0.0001**
65+	55.61% (42,130)	38.59% (16,258)				Ref		Ref	
Race/ethnicity			<0.0001						
NH White	51.59% (39,085)	43.72% (17,087)				Ref		Ref	
NH Black	13.13% (9949)	45.06% (4483)		—	—	1.005	0.8441	1.012	0.6222
Latino	21.60% (16,364)	47.32% (7743)		—	—	1.030	0.1715	1.027	0.2240
NH Others	13.68% (10,363)	36.26% (3758)		—	—	**0.677**	**<0.0001**	**0.672**	**<0.0001**
Anatomical site			<0.0001						
Cardia	31.71% (24,026)	42.48% (10,206)				Ref		Ref	
Non‐cardia	45.76% (34,665)	37.84% (13,117)		—	—	**0.888**	**<0.0001**	**0.890**	**<0.0001**
Overlap	7.50% (5680)	51.36% (2917)		—	—	**1.481**	**<0.0001**	**1.483**	**<0.0001**
NOS	15.03% (11,390)	59.97% (6831)		—	—	**2.170**	**<0.0001**	**2.174**	**<0.0001**
Year of DX			<0.0001						
2004–2007	30.90% (22,799)	42.13% (9606)				Ref		Ref	
2008–2011	30.05% (22,765)	42.91% (9769)		—	—	1.032	0.1016	1.033	0.0980
2012–2016	39.86% (30,197)	45.36% (13,696)		—	—	**1.152**	**<0.0001**	**1.152**	**<0.0001**
County‐level indicators mean (Std)									
% Smokers	14.104 (3.392)	14.085 (3.386)	0.1575	—	—	—	—	**0.937**	**0.0002**
% Obese	26.064 (4.915)	26.096 (4.895)	0.1081	—	—	—	—	1.031	0.0971
% Excessive alcohol	18.446 (2.350)	18.440 (2.339)	0.5534	—	—	—	—	1.008	0.4667
Food environment index	7.937 (0.865)	7.930 (0.856)	0.0383	—	—	—	—	0.989	0.4315
Social deprivation index			0.1682						
SDI 0–20 (least deprived)	14.32% (10,851)	42.99% (4,665)						Ref	
SDI 21–79	49.12% (37,213)	43.96% (16,357)		—	—	—	—	1.047	0.1426
SDI 80–100 (most deprived)	36.56% (27,697)	43.50% (12,049)		—	—	—	—	0.998	0.7603

Bold values are significant at *p* < 0.05 or less.

Adjusted for Reporting Source.

Abbreviations: DX, Diagnosis; GCA, Gastric Adenocarcinoma.

**TABLE 2:** Logistic regression models for late‐stage GCA diagnosis by location, adults 18–89, 2004–2016

*n*	SEER		Texas		Texas w/o STX		STX	
	64,758		11,003		8663		2340	
	OR	*p* value	OR	*p* value	OR	*p* value	OR	*p* value
Sex								
Female	Ref		Ref		Ref		Ref	
Male	1.017	0.3233	1.021	0.6283	1.031	0.5225	0.991	0.9205
Age at DX								
20–39	**2.432**	**<0.0001**	**2.666**	**<0.0001**	**2.426**	**<0.0001**	**3.896**	**<0.0001**
40–64	**1.488**	**<0.0001**	**1.668**	**<0.0001**	**1.595**	**<0.0001**	**1.979**	**<0.0001**
65+	Ref		Ref		Ref		Ref	
Race/ethnicity								
NH White	Ref		Ref		Ref		Ref	
NH Black	1.023	0.4086	0.969	0.6308	0.993	0.9185	0.779	0.3723
Latino	1.017	0.5057	1.055	0.3039	1.092	0.1296	1.002	0.9877
NH others	**0.664**	**<0.0001**	**0.781**	**0.0101**	**0.789**	**0.0159**	1.058	0.9010
Anatomical site								
Cardia	Ref		Ref		Ref		Ref	
Non‐cardia	**0.890**	**<0.0001**	**0.875**	**0.0105**	**0.858**	**0.0078**	0.957	0.7290
Overlap	**1.509**	**<0.0001**	**1.311**	**0.0013**	**1.373**	**0.0010**	1.209	0.2945
NOS	**2.270**	**<0.0001**	**1.798**	**<0.0001**	**1.738**	**<0.0001**	**2.003**	**<0.0001**
Year of DX								
2004–2007	Ref		Ref		Ref		Ref	
2008–2011	1.033	0.1255	1.031	0.5451	1.009	0.8719	1.155	0.2014
2012–2016	**1.139**	**<0.0001**	**1.231**	**<0.0001**	**1.194**	**0.0009**	**1.431**	**0.0010**
County‐level indicators								
% Smokers (z‐score)	**0.926**	**<0.0001**	1.017	0.8162	0.888	0.0818	**1.423**	**0.0197**
% Obese (z‐score)	1.039	0.0533	0.958	0.4265	0.987	0.7954	0.992	0.9512
% Excessive alcohol (z‐score)	1.010	0.4121	1.069	0.1236	1.021	0.6443	1.152	0.1102
Food environment index (z‐score)	0.978	0.1395	1.006	0.8600	0.988	0.7362	1.080	0.4608
Social deprivation index								
SDI 0–20 (least deprived)	Ref		Ref		Ref		Ref	
SDI 21–79	1.037	0.2815	1.153	0.1338	**1.247**	**0.0073**	0.765	0.2661
SDI 80–100 (most deprived)	0.959	0.3262	1.160	0.1793	**1.198**	**0.0280**	0.795	0.4608

Bold values are significant at *p* < 0.05 or less.

Also adjusted for reporting source.

**TABLE 3:** Logistic regression models for late‐stage GCA diagnosis by anatomic site, adults 18–89, 2004–2016

*n*	Cardia		Non‐cardia		Overlap		NOS	
	24,026		34,665		5680		11,390	
	OR	*p* value	OR	*p* value	OR	*p* value	OR	*p* value
Location								
SEER	Ref		Ref		Ref		Ref	
TX (w/o) STX	1.033	0.5204	0.934	0.1946	0.946	0.6035	**0.782**	**0.0018**
STX	**0.755**	**0.0066**	0.893	0.1832	**0.591**	**0.0014**	**0.709**	**0.0082**
Sex								
Female	Ref		Ref		Ref		Ref	
Male	0.986	0.6718	**1.070**	**0.0029**	1.052	0.3541	0.935	0.0929
Age at DX								
20–39	**2.171**	**<0.0001**	**2.615**	**<0.0001**	**2.404**	**<0.0001**	**2.691**	**<0.0001**
40–64	**1.359**	**<0.0001**	**1.565**	**<0.0001**	**1.553**	**<0.0001**	**1.781**	**<0.0001**
65+	Ref		Ref		Ref		Ref	
Race/ethnicity								
NH White	Ref		Ref		Ref		Ref	
NH Black	**1.285**	**<0.0001**	0.943	0.0888	0.982	0.8279	**0.887**	**0.0450**
Latino	**1.128**	**0.0079**	0.965	0.2602	1.110	0.1577	0.915	0.1070
NH others	**0.854**	**0.0076**	**0.619**	**<0.0001**	**0.813**	**0.0163**	**0.560**	**<0.0001**
Year of DX								
2004–2007	Ref		Ref		Ref		Ref	
2008–2011	1.050	0.1653	1.016	0.5722	0.999	0.9909	1.064	0.2041
2012–2016	**1.186**	**<0.0001**	**1.085**	**0.0027**	1.057	0.3077	**1.355**	**<0.0001**
County‐level indicators								
% Smokers (z‐score)	**0.945**	**0.0193**	0.952	0.0637	**0.886**	**0.0295**	**0.858**	**0.0005**
% Obese (z‐score)	1.045	0.0748	1.018	0.5072	1.105	0.0758	1.014	0.7620
% Excessive alcohol (z‐score)	0.996	0.7948	1.033	0.0508	1.050	0.1412	0.959	0.1299
Food environment index (z‐score)	1.013	0.5184	0.964	0.0699	1.013	0.7654	0.977	0.4604
Social deprivation index								
SDI 0–20 (least deprived)	Ref		Ref		Ref		Ref	
SDI 21–79	1.029	0.4675	1.042	0.3874	1.008	0.9325	**1.212**	**0.0126**
SDI 80–100 (most deprived)	1.000	0.9922	1.002	0.9783	0.999	0.9899	1.070	0.4481

Bold values are significant at *p* < 0.05 or less.

Also adjusted for reporting source.

Because the combined Summary Stage 2000 variable in the SEER and TCR used to define our late‐stage outcome contained cases with unknown stage (10.19% of sample excluded from the analysis), we conducted sensitivity analyses including patients with unknown stage at diagnosis as part of the reference group (i.e., non‐late‐stage disease). Since SDI data are based on ACS data collected during 2011–2015, we also conducted a sensitivity analysis using cases limited to that year range. We conducted all descriptive statistics, multilevel modeling, and sensitivity analyses for late‐stage GCA diagnosis using SAS 9.4. We considered all results significant if *α* ≤ 0.05.

## 3 RESULTS

### 3.1 Frequencies and incidence

For incidence analyses, we had over 117,400 cases of primary invasive GCA diagnosed from 2004 to 2016 in the United States; 20,418 GCA cases registered in TX; and 4192 in STX. Overall GCA incidence rates were significantly higher in Latinos than NHWs in all three regions (Table S1). GCA was more common in males than females in all regions and both race/ethnicity groups. Latinos had higher rates of non‐cardia, overlap, and NOS GCA rates in all three regions, with the greatest ethnic disparity seen in STX Latino versus NHW men for overlap GCA (RR 4.39; 95% CI: 2.85, 6.93). NHWs had significantly higher rates of cardia GCA than Latinos in all three regions except for women in all regions, where there was no ethnic difference.

### 3.2 Descriptive statistics of late‐stage gastric cancer diagnosis

For the more granular analyses of late‐stage disease, our sample consisted of over 75,700 cases of primary invasive GCA diagnosed from 2004 to 2016 in the United States; 8663 GCA cases registered in TX, not counting STX; and 2340 in STX. (Table 1). Approximately 85% of patients came from SEER and 15% from TCR. Approximately 3% of patients lived in STX at the time of diagnosis. The sample was majority male (64%), age 65 or older (55.6%), and NHW (51.6%). About 40% of patients were diagnosed between 2012 and 2016. Most cases were reported by inpatient/outpatient hospitals or clinics (94.8%; data not shown). Approximately 44% of patients were diagnosed with late‐stage GCA.

### 3.3 Multilevel logistic regression

In the Model 1 adjusted for reporting source, patients from TX had higher, and STX lower odds of having late‐stage GCA compared to SEER patients, but these were not significant (OR: 1.04 and 0.90, *p* = 0.18 and 0.07) (Table 1). Adjusting for patient characteristics reduced the odds of late‐stage GCA for TX and STX patients, with STX being significantly lower (OR: 0.81, *p* = 0.001, Model 2) compared to SEER. Furthermore, younger patients, those with overlapping and NOS lesions and those diagnosed during 2012–2016 had significantly higher odds of late‐stage diagnosis than patients aged 65+, those with cardia GCA, and those diagnosed during 2004–2007, respectively. NH Others had lower odds of late‐stage disease than NHW (OR: 0.68, *p* < 0.001). Adjusting for county‐level variables, patients from TX and STX had significantly lower odds of being diagnosed with late‐stage GCA compared to SEER patients (OR: 0.91 and 0.81, *p* = 0.02 and 0.001, Model 3). Patients living in counties with higher proportions of smokers at the time of diagnosis also had lower odds of late‐stage diagnosis (OR: 0.94, *p* = 0.0002); meaning that for every standard deviation increase in county‐level smoking prevalence, the odds of being diagnosed with late‐stage disease decreased by 6%. County‐level SDI did not significantly impact patients’ odds of late‐stage GCA.

We saw some different effects in models stratified by location (Table 2). Latinos were slightly more likely to have late‐stage disease compared to NHWs only in TX without STX (OR: 1.09, *p* = 0.13). Overlap and NOS lesions had higher odds compared to cardia in all locations, except overlap in STX (OR: 1.21, *p* = 0.29). In TX without STX, living in a county with medium and high SDI were associated with increased odds of late‐stage GCA compared to counties with the lowest SDI (i.e., least deprived counties) (OR: 1.25 and 1.20, *p* = 0.007 and 0.028). In STX, patients from counties with higher smoking had greater odds of late‐stage GCA (OR: 1.42, *p* = 0.02); meaning that for every standard deviation increase in county‐level smoking prevalence, the odds of being diagnosed with late‐stage disease increased by 42%. The opposite was true in SEER (OR: 0.93, *p* < 0.001); for every standard deviation increase in county‐level smoking prevalence, the odds of being diagnosed with late‐stage disease decreased by 7%. Although younger patients had consistently higher odds of late‐stage GCA in all analyses, in STX the age 20–39 group was almost four times as likely to have a late‐stage diagnosis compared to 65+ year olds (OR: 3.9, *p* < 0.001).

In models stratified by anatomic site, Latinos and NH blacks with cardia GCA were more likely to have late‐stage GCA than NHWs (OR: 1.13 and 1.29, *p* = 0.008 and <0.001) (Table 3). Patients living in counties with higher smoking prevalence had lower odds of late‐stage cardia (OR: 0.94, *p* = 0.019), overlapping (OR: 0.89, *p* = 0.03), and NOS (OR: 0.86, *p* = 0.001) lesions. In STX, patients with NOS GCA and who lived in medium SDI counties were associated with higher odds of being diagnosed with late‐stage GCA (OR: 1.21, *p* = 0.013).

Sensitivity analyses including patients with unknown stage as part of the reference group (10.19% of the sample) showed similar results as for the main analysis (Tables [Link], [Link], [Link]). However, there were additional findings: males had significantly higher odds of having late‐stage disease in the full model (Table S2), in the stratified analysis for SEER (Table S3), and the stratified analyses for patients with cardia and non‐cardia GCA (Table S4).

Sensitivity analyses limited to the years 2011–2015 (Tables [Link], [Link], [Link]) supported the main findings for individual‐level characteristics and confirmed the association of county‐level smoking prevalence with late‐stage disease in the STX population (OR: 1.753, *p* = 0.0231).

## 4 DISCUSSION

Our study provides an overview of GCA rates and late‐stage diagnosis in the United States, Texas, and South Texas (STX) from 2004 to 2016 among Latinos and non‐Latinos. Texas's exclusion from SEER data has limited previous comparative assessments of GCA incidence rates. Although Camargo and colleagues’ study reported robust data including Texas, only 7.9% of SEER‐9 participants were Latinos.^16^ Previous studies were limited by region (El Paso County vs. Texas)^19^ or population examined (predominantly male veterans).^29^ The present study used both SEER‐21 and Texas Cancer Registry data, ensuring adequate representation of the standard United States, TX, and STX populations.

Consistent with prior studies,^7^, ^17^, ^20^ we found that overall GCA incidence rates in Texas and STX were higher in Latinos than in NHWs, despite lower frequencies in the state and STX region compared to the United States. Cardia GCA was more common in NHWs, contrasting with non‐cardia, overlap, and not otherwise specified (NOS) in Latinos. This was also consistent with prior studies.^19^, ^30^, ^31^ An exception we found was in cardia GCA among women, which had similar rates among NHWs and Latinos. Although GC overall is a predominantly male disease, our findings indicate that additional measures are needed to target women who may be at risk for cardia GCA. Further research should identify such measures, as well as specific populations of women at higher risk.

Non‐cardia, overlap, and NOS GCA were disproportionately represented among Latinos in all regions compared to non‐Latinos. The high *H. pylori* infection rate in Latinos^32^, ^33^ coupled with *H. pylori* being the most well‐known risk factor for more distal stomach cancers,^34^ may account for this disparity.

In contrast to overall GCA, late‐stage diagnosis odds were significantly higher in the United States than both Texas (not including STX) and STX. Texas providers may screen earlier due to the higher overall GCA prevalence in Texas and STX. This possibility can be explored in future studies. Furthermore, younger patients, those with overlapping and NOS lesions and those diagnosed during 2012–2016 had significantly higher odds of late‐stage diagnosis than patients aged 65+, those with cardia GCA, and those diagnosed during 2004–2007, respectively. The increase in younger patients with GCA partially reflects previous studies, though these did not focus on late‐stage disease.^8^, ^9^, ^10^ To the best of our knowledge, we are the first to report this finding. This along with the increased late‐stage diagnoses of overlap and NOS lesions is a disturbing indication of the lack of standardized screening practices for GC, unlike those for colorectal, breast, and ovarian cancers. The guidelines for surveillance of precursor conditions like intestinal metaplasia are based on predominantly low‐quality evidence,^35^ and only symptomatic patients are recommended to be tested for *H. pylori* in lower prevalence countries like the United States.^34^ Our results support the need for development of effective methodologies of early GC screening in the United States.^36^

None of the county‐level risk factors contributed to higher odds of late‐stage disease in the main analysis. Smoking was associated with decreased odds of late‐stage disease in the main and anatomic site analyses. While counterintuitive, this is consistent with previous studies,^1^, ^6^ including a recent population‐based cohort study of SEER data from 2007 to 2015 which found that Latinos with non‐cardia GC were less likely to reside in counties with high smoking prevalence.^9^ As stated by Gnaldi and colleagues, in the case of disagreement between results from studies conducted at different levels, additional research is needed before it can be concluded that estimates including ecological variables are inappropriate.^37^ It is also possible that smokers are screened earlier and thus their disease is caught earlier. However, confirming this is beyond the scope of this paper. In contrast, in the model stratified by location, STX smokers had a significantly increased risk of late‐stage GCA. This is not explained by the current smoking prevalence rates in STX and TX counties, which are significantly lower than the nation (15.7% vs. 17.7%, *p* < 0.001; data not shown).

We explored socioeconomic status's connection to late‐stage GCA using the SDI, a more complex measure than poverty alone. For the full model, SDI was not significantly associated with late‐stage disease, but in the stratified model by location, only in TX without STX patients living in counties with medium and high SDI were associated with increased odds of late‐stage GCA compared to those with the lowest SDI. This was surprising, given the high poverty rate in STX (~22% below 150% of the federal poverty line in 2018 vs. 12% in the United States). In contrast, in the model stratified by anatomic site, living in a county with medium SDI in STX only was associated with increased odds of NOS late‐stage GCA specifically. Additional studies, preferably using individual‐level data, are required to adequately characterize the interplay of SDI with *H. pylori* infection, which has been linked to lower socioeconomic class,^38^ in TX and STX.

The lack of significant county‐level behavioral results for late‐stage disease, except for smoking, in STX is surprising, considering the high proportion of Latino residents and high prevalence of relevant risk factors. This may be due to the smaller sample size. Underdiagnosis is also a possibility, which must be further pursued.

The stratification of our data by anatomic site (e.g., cardia, non‐cardia) is consistent with findings from The Cancer Genome Atlas molecular classification of GCAs.^39^ The inclusion of overlapping and NOS GCA lesions as separate categories is justified, given the significant disparities seen across all regions, and to the best of our knowledge is the first analysis of these anatomic sites.

One strength of this study lies in extracting data from both SEER and TCR, enabling us to obtain precise rates and risk estimations in all geographic regions analyzed with the same methodology, making relevant comparisons between populations possible. Another strength is our multilevel approach using county‐level GCA risk factors, including smoking, obesity, excessive alcohol consumption, food environment, and social deprivation. This allowed for a richer comparison between groups. However, one limitation is that both registries consider Latinos as a single group, which prevented us from identifying differences by ethnic subgroups. However, 88% of Texas Latinos are of Mexican origin, so data from Texas and STX may well represent this specific Latino subgroup.^11^ Additionally, SEER and NAACCR do not have identical completeness requirements and thus have differences in organization. This may account for histology differences observed. County‐level indicators (SDI, CHR variables) may not accurately describe individual‐level living conditions and risk factors and warrant careful interpretation as they can lead to the ecological fallacy. While previous research has shown that smaller area estimates are better proxies for individual‐level characteristics,^40^, ^41^ using county‐level indicators are a first step in identifying additional factors not typically included in registry data. Further examination of our findings based on county‐level variables is warranted, ideally with individual‐level data. Additionally, county‐level variables were measured at one time point only, which may not overlap with patients’ year of diagnosis. We conducted sensitivity analyses to address the limitation of county‐level variables to the years 2011–2015, the range for which the SDI is available. Our findings regarding smoking prevalence for patients in STX was confirmed with the significantly reduced sample. Our sample contained approximately 11% of patients with unknown stage, who we excluded from our main analysis. As part of our sensitivity analysis, we included those with unknown stage as part of the reference group in our late‐stage outcome variable. TCR had higher rates of unknown stage than SEER (14.1% vs. 9.5%), and in a previous study the type of the reporting source predicted the unknown stage.^28^ We included reporting source in all analyses to account for reporting differences. Our sensitivity analyses including unknown stage in the analytic sample produced similar results.

As the US Latino population continues to grow, the higher GCA incidence in Latinos, particularly of late‐stage disease, which is less responsive to therapy, is an increasingly important public health concern. Future studies should include genetic factors, which was beyond the scope of this paper, and interactions between alcohol consumption and social deprivation as contributors to late‐stage GCA.

## CONFLICT OF INTEREST

The authors declare that they have no conflict of interest.

## Supporting information

Table S1

Table S2

Table S3

Table S4

Table S5

Table S6

Table S7

## Data Availability

The data that support the findings of this study are available from the corresponding author upon reasonable request.
