# Supplementary material for: Gastric adenocarcinoma burden and late‐stage diagnosis in Latino and non‐Latino populations in the United States and Texas, during 2004–2016: A multilevel analysis
Source: Cancer Med. 2021 Aug 19;10(18):6468–79. doi: 10.1002/cam4.4175 (PMC8446571; doi:10.1002/cam4.4175)
Supplement: Supplementary file 1 — Table S1 [file CAM4-10-6468-s006.docx]

Supplemental Table 1. Incidence rates^a^ and Rate Ratios^b^ (RR) of GCA by type among 20+ years-old Latinos and non-Hispanic Whites (NHW) from US, Texas, and South Texas, 2004-2016

|  | **Cardia** | | | **Non-cardia** | | | **Overlap** | | | **NOS** | | | **Total** | | | |
| --- | --- | --- | --- | --- | --- | --- | --- | --- | --- | --- | --- | --- | --- | --- | --- | --- |
|  | **Cases** | **Rate**  **(95% CI)** | **RR**  **(95% CI)** | **Cases** | **Rate**  **(95% CI)** | **RR**  **(95% CI)** | **Cases** | **Rate**  **(95% CI)** | **RR**  **(95% CI)** | **Cases** | **Rate**  **(95% CI)** | **RR**  **(95% CI)** | **Cases** | **Rate**  **(95% CI)** | **RR**  **(95% CI)** | **Person-years (Millions)** |
| **US SEER** |  |  |  |  |  |  |  |  |  |  |  |  |  |  |  |  |
| **Total**^a^ | 33,097 | 2.97  (2.93, 3.00) |  | 54,256 | 4.93  (4.89, 4.98) |  | 7,906 | 0.72  (0.70, 0.74) |  | 22,199 | 2.01  (1.98, 2.04) |  | 117,458 | 10.63  (10.57, 10.69) |  | 1,079 |
| NHW | 26,143 | 3.31  (3.27, 3.35) | Ref. | 24,777 | 3.14  (3.10, 3.18) | Ref. | 3,706 | 0.47  (0.46, 0.49) | Ref. | 11,392 | 1.44  (1.42, 1.47) | Ref. | 66,018 | 8.37  (8.31, 8.44) | Ref. | 655 |
| Latino | 2,878 | 2.28  (2.20, 2.37) | 0.69  (0.66, 0.72) | 10,478 | 8.52  (8.35, 8.69) | 2.71  (2.64, 2.77) | 1,766 | 1.39  (1.32, 1.46) | 2.93  (2.76, 3.11) | 4,102 | 3.25  (3.15, 3.36) | 2.26  (2.17, 2.34) | 19,224 | 15.44  (15.21, 15.67) | 1.84  (1.81, 1.88) | 194 |
| **Males** | 25,423 | 5.10  (5.03, 5.16) |  | 29,518 | 6.14  (6.07, 6.21) |  | 4,486 | 0.92  (0.90, 0.95) |  | 11,766 | 2.44  (2.40, 2.49) |  | 71,193 | 14.60  (14.49, 14.71) |  | 522 |
| NHW | 20,524 | 5.73  (5.65, 5.81) | Ref. | 13,355 | 3.89  (3.82, 3.96) | Ref. | 2,094 | 0.60  (0.58, 0.63) | Ref. | 6,069 | 1.77  (1.72, 1.81) | Ref. | 42,042 | 11.99  (11.88, 12.11) | Ref. | 318 |
| Latino | 2,030 | 3.67  (3.49, 3.84) | 0.64  (0.61, 0.67) | 5,648 | 10.56  (10.26, 10.87) | 2.71  (2.62, 2.81) | 1,024 | 1.90  (1.77, 2.03) | 3.14  (2.90, 3.41) | 2,104 | 3.86  (3.68, 4.05) | 2.19  (2.07, 2.31) | 10,806 | 19.99  (19.58, 20.40) | 1.67  (1.63, 1.70) | 97 |
| **Females** | 7,674 | 1.25  (1.22, 1.27) |  | 24,738 | 4.04  (3.99, 4.09) |  | 3,420 | 0.56  (0.55, 0.58) |  | 10,433 | 1.69  (1.66, 1.72) |  | 46,265 | 7.54  (7.47, 7.61) |  | 557 |
| NHW | 5,619 | 1.29  (1.25, 1.32) | Ref. | 11,422 | 2.60  (2.55, 2.65) | Ref. | 1,612 | 0.37  (0.35, 0.39) | Ref. | 5,323 | 1.20  (1.17, 1.24) | Ref. | 23,976 | 5.46  (5.39, 5.54) | Ref. | 337 |
| Latino | 848 | 1.22  (1.13, 1.30) | 0.94  (0.87, 1.02) | 4,830 | 7.03  (6.83, 7.24) | 2.70  (2.61, 2.80) | 742 | 1.02  (0.95, 1.10) | 2.75  (2.51, 3.02) | 1,998 | 2.83  (2.70, 2.96) | 2.35  (2.23, 2.48) | 8,418 | 12.10  (11.83, 12.37) | 2.21  (2.16, 2.27) | 97 |
| **TEXAS** |  |  |  |  |  |  |  |  |  |  |  |  |  |  |  |  |
| **Total** | 5,003 | 2.32  (2.25, 2.39) |  | 8,256 | 3.89  (3.81, 3.98) |  | 1,218 | 0.57  (0.54, 0.61) |  | 5,941 | 2.82  (2.75, 2.89) |  | 20,418 | 9.60  (9.47, 9.74) |  | 229 |
| NHW | 3,617 | 2.66  (2.57, 2.75) | Ref. | 2,752 | 2.06  (1.98, 2.14) | Ref. | 398 | 0.30  (0.27, 0.33) | Ref. | 2,400 | 1.82  (1.74, 1.89) | Ref. | 9,167 | 6.83  (6.69, 6.97) | Ref. | 116 |
| Latino | 945 | 1.84  (1.72, 1.97) | 0.69  (0.64, 0.75) | 3,515 | 7.14  (6.90, 7.40) | 3.47  (3.30, 3.66) | 556 | 1.09  (1.00, 1.19) | 3.65  (3.19, 4.17) | 2,307 | 4.59  (4.40, 4.79) | 2.53  (2.38, 2.68) | 7,323 | 14.67  (14.32, 15.03) | 2.15  (2.08, 2.22) | 76 |
| **Males** | 3,875 | 3.94  (3.81, 4.07) |  | 4,621 | 4.86  (4.72, 5.01) |  | 711 | 0.74  (0.68, 0.79) |  | 3,094 | 3.31  (3.19, 3.43) |  | 12,301 | 12.84  (12.61, 13.08) |  | 112 |
| NHW | 2,900 | 4.59  (4.42, 4.76) | Ref. | 1,495 | 2.48  (2.35, 2.61) | Ref. | 229 | 0.38  (0.33, 0.43) | Ref. | 1,256 | 2.13  (2.01, 2.25) | Ref. | 5,880 | 9.57  (9.33, 9.83) | Ref. | 57 |
| Latino | 667 | 2.90  (2.67, 3.15) | 0.63  (0.58, 0.69) | 1,926 | 8.95  (8.52, 9.39) | 3.61  (3.36, 3.88) | 321 | 1.43  (1.26, 1.60) | 3.77  (3.14, 4.52) | 1,180 | 5.49  (5.16, 5.85) | 2.58  (2.37, 2.81) | 4,094 | 18.77  (18.14, 19.40) | 1.96  (1.88, 2.05) | 38 |
| **Females** | 1,128 | 0.98  (0.92, 1.03) |  | 3,635 | 3.16  (3.05, 3.26) |  | 507 | 0.44  (0.40, 0.48) |  | 2,847 | 2.46  (2.37, 2.56) |  | 8,117 | 7.03  (6.88, 7.19) |  | 117 |
| NHW | 717 | 0.99  (0.92, 1.07) | Ref. | 1,257 | 1.74  (1.64, 1.84) | Ref. | 169 | 0.24  (0.20, 0.27) | Ref. | 1,144 | 1.59  (1.49, 1.68) | Ref. | 3,287 | 4.55  (4.40, 4.72) | Ref. | 59 |
| Latino | 278 | 1.01  (0.89, 1.14) | 1.01  (0.88, 1.17) | 1,589 | 5.82  (5.53, 6.12) | 3.35  (3.10, 3.61) | 235 | 0.83  (0.73, 0.95) | 3.54  (2.88, 4.36) | 1,127 | 3.98  (3.75, 4.23) | 2.51  (2.31, 2.74) | 3,229 | 11.64  (11.23, 12.06) | 2.56  (2.43, 2.69) | 38 |
| **SOUTH TEXAS** |  |  |  |  |  |  |  |  |  |  |  |  |  |  |  |  |
| **Total** | 791 | 2.04  (1.90, 2.19) |  | 1,951 | 5.10  (4.87, 5.33) |  | 306 | 0.80  (0.72, 0.90) |  | 1,144 | 2.97  (2.80, 3.15) |  | 4,192 | 10.91  (10.58, 11.25) |  | 40 |
| NHW | 397 | 2.46  (2.22, 2.73) | Ref. | 315 | 1.98  (1.76, 2.22) | Ref. | 52 | 0.32  (0.24, 0.42) | Ref. | 226 | 1.43  (1.25, 1.64) | Ref. | 990 | 6.19  (5.80, 6.60) | Ref. | 12 |
| Latino | 373 | 1.79  (1.61, 1.99) | 0.73  (0.63, 0.84) | 1,535 | 7.48  (7.10, 7.87) | 3.78  (3.33, 4.29) | 240 | 1.16  (1.02, 1.32) | 3.67  (2.68, 5.08) | 855 | 4.12  (3.84, 4.41) | 2.87  (2.47, 3.35) | 3,003 | 14.55  (14.02, 15.09) | 2.35  (2.18, 2.53) | 25 |
| **Males** | 591 | 3.40  (3.13, 3.69) |  | 1,076 | 6.30  (5.92, 6.69) |  | 175 | 1.02  (0.87, 1.18) |  | 611 | 3.58  (3.30, 3.88) |  | 2,453 | 14.30  (13.73, 14.88) |  | 19 |
| NHW | 318 | 4.17  (3.72, 4.67) | Ref. | 173 | 2.35  (2.01, 2.74) | Ref. | 27 | 0.35  (0.23, 0.52) | Ref. | 125 | 1.73  (1.44, 2.08) | Ref. | 643 | 8.61  (7.94, 9.31) | Ref. | 6 |
| Latino | 260 | 2.89  (2.54, 3.28) | 0.69  (0.58, 0.82) | 838 | 9.54  (8.88, 10.23) | 4.06  (3.43, 4.83) | 139 | 1.55  (1.29, 1.84) | 4.39  (2.85, 6.93) | 454 | 5.18  (4.69, 5.69) | 2.99  (2.43, 3.69) | 1,691 | 19.16  (18.22, 20.13) | 2.23  (2.03, 2.45) | 12 |
| **Females** | 200 | 0.94  (0.82, 1.08) |  | 875 | 4.20  (3.92, 4.49) |  | 131 | 0.62  (0.52, 0.74) |  | 533 | 2.51  (2.30, 2.74) |  | 1,739 | 8.28  (7.89, 8.68) |  | 20 |
| NHW | 79 | 0.94  (0.74, 1.19) | Ref. | 142 | 1.71  (1.43, 2.03) | Ref. | 25 | 0.28  (0.18, 0.43) | Ref. | 101 | 1.18  (0.96, 1.45) | Ref. | 347 | 4.12  (3.68, 4.60) | Ref. | 6 |
| Latino | 113 | 0.97  (0.79, 1.16) | 1.02  (0.75, 1.39) | 697 | 6.02  (5.58, 6.49) | 3.52  (2.91, 4.27) | 101 | 0.87  (0.71, 1.06) | 3.08  (1.92, 5.07) | 401 | 3.38  (3.05, 3.73) | 2.86  (2.27, 3.62) | 1,312 | 11.24  (10.63, 11.87) | 2.73  (2.41, 3.09) | 13 |

^a^Rates per 100,00 and age-adjusted to 2000 US Standard Population (19 age groups).

^b^Rate Ratios calculated using NHW group as reference. All RRs are significant at p<0.05 except for Female cardia GCA in SEER, TEXAS and SOUTH TEXAS.

^c^All totals (bold) include all races and ethnicities.
