# Supplementary material for: Gastric adenocarcinoma burden and late‐stage diagnosis in Latino and non‐Latino populations in the United States and Texas, during 2004–2016: A multilevel analysis
Source: Cancer Med. 2021 Aug 19;10(18):6468–79. doi: 10.1002/cam4.4175 (PMC8446571; doi:10.1002/cam4.4175)
Supplement: Supplementary file 2 — Table S2 [file CAM4-10-6468-s001.docx]

| Supplemental Table 2: Descriptive Statistics and Multilevel Logistic Regression Models for Late-stage GCA Diagnosis including Unknown Stage, Adults 20+, 2004-2016 | | | | | | | | | |
| --- | --- | --- | --- | --- | --- | --- | --- | --- | --- |
|  | **Descriptive Statistics** | | | **Logistic Regression Models** | | | | | |
|  | **All GCA Patients** | **Patients with Late-Stage GCA DX** | | **Model 1** | | **Model 2** | | **Model 3** | |
| **n** | 84,359 | 33,071 | | 84,359 | | 84,359 | | 84,359 | |
|  | **% (n)** | **% (n)** | **p-value** | **OR** | **p-value** | **OR** | **p-value** | **OR** | **p-value** |
| **Late-Stage GCA DX** |  |  | --- |  |  |  |  |  |  |
| Yes | 39.20% (33,071) | --- |  | --- | --- | --- | --- | --- | --- |
| No | 60.80% (51,288) | --- |  | --- | --- | --- | --- | --- | --- |
| **Location** |  |  | <0.0001 |  |  |  |  |  |  |
| SEER | 84.82% (71,552) | 39.37% (28,170) |  | Ref |  | Ref |  | Ref |  |
| TX (w/o) STX | 11.92% (10,053) | 39.39% (3,960) |  | **0.925** | **0.0114** | **0.842** | **<0.0001** | **0.814** | **<0.0001** |
| STX | 3.26% (2,754) | 34.17% (941) |  | **0.781** | **<0.0001** | **0.703** | **<0.0001** | **0.700** | **<0.0001** |
| **Sex** |  |  | <0.0001 |  |  |  |  |  |  |
| Female | 37.16% (31,345) | 38.18% (11,967) |  |  |  | Ref |  | Ref |  |
| Male | 62.82% (53,014) | 39.81% (21,104) |  | --- | --- | **1.049** | **0.0020** | **1.049** | **0.0017** |
| **Age at DX** |  |  | <0.0001 |  |  |  |  |  |  |
| 20-39 | 3.83% (3,231) | 58.16% (1,879) |  | --- | --- | **2.677** | **<0.0001** | **2.678** | **<0.0001** |
| 40-64 | 38.53% (32,506) | 45.94% (14,934) |  | --- | --- | **1.675** | **<0.0001** | **1.676** | **<0.0001** |
| 65+ | 57.64% (48,622) | 33.44% (16,258) |  |  |  | Ref |  | Ref |  |
| **Race/Ethnicity** |  |  | <0.0001 |  |  |  |  |  |  |
| NH White | 51.59% (43,517) | 39.27% (17,087) |  |  |  | Ref |  | Ref |  |
| NH Black | 13.16% (11,101) | 40,38% (4,483) |  | --- | --- | 1.005 | 0.8408 | 1.011 | 0.6647 |
| Latino | 21.51% (18,147) | 42.67% (7,743) |  | --- | --- | **1.047** | **0.0281** | **1.044** | **0.0418** |
| NH Others | 13.74% (11,594) | 32.41% (3,758) |  | --- | --- | **0.690** | **<0.0001** | **0.684** | **<0.0001** |
| **Anatomical Site** |  |  | <0.0001 |  |  |  |  |  |  |
| Cardia | 31.02% (26,170) | 39.00% (10,206) |  |  |  | Ref |  | Ref |  |
| Non-Cardia | 45.01% (37,971) | 34.54% (13,117) |  | --- | --- | **0.896** | **<0.0001** | **0.898** | **<0.0001** |
| Overlap | 7.24% (6,106) | 47.77% (2,917) |  | --- | --- | **1.481** | **<0.0001** | **1.483** | **<0.0001** |
| NOS | 16.73% (14,112) | 48.41% (6,831) |  | --- | --- | **1.641** | **<0.0001** | **1.643** | **<0.0001** |
| **Year of DX** |  |  | <0.0001 |  |  |  |  |  |  |
| 2004-2007 | 30.25% (25,519) | 37.64% (9,606) |  |  |  | Ref |  | Ref |  |
| 2008-2011 | 30.11% (25,399) | 38.46% (9,769) |  | --- | --- | 1.032 | 0.0926 | 1.032 | 0.0891 |
| 2012-2016 | 39.64% (33,441) | 40.94% (13,696) |  | --- | --- | **1.153** | **<0.0001** | **1.153** | **<0.0001** |
| **County Level Indicators Mean (Std)** |  |  |  |  |  |  |  |  |  |
| % Smokers | 14.108 (3.375) | 14.085 (3.386) | 0.1110 | --- | --- | --- | --- | **0.933** | **<0.0001** |
| % Obese | 26.076 (4.898) | 26.096 (4.895) | 0.3277 | --- | --- | --- | --- | 1.028 | 0.1237 |
| % Excessive Alcohol | 18.108 (2.338) | 18.440 (2.339) | 0.5222 | --- | --- | --- | --- | 1.009 | 0.4485 |
| Food Environment Index | 7.935 (0.867) | 7.930 (0.856) | 0.2274 | --- | --- | --- | --- | 0.980 | 0.1377 |
| **Social Deprivation Index** |  |  | 0.0004 |  |  |  |  |  |  |
| SDI 0-20 (least deprived) | 14.29% (12,057) | 38.69% (4,665) |  |  |  |  |  | Ref |  |
| SDI 21-79 | 48.62% (41,016) | 39.88% (16,357) |  | --- | --- | --- | --- | 1.049 | 0.1242 |
| SDI 80-100 (most deprived) | 37.09% (31,286) | 38.51% (12,049) |  | --- | --- | --- | --- | 0.985 | 0.6890 |

Adjusted for Reporting Source. GCA: Gastric Adenocarcinoma; DX: Diagnosis
