# Supplementary material for: Gastric adenocarcinoma burden and late‐stage diagnosis in Latino and non‐Latino populations in the United States and Texas, during 2004–2016: A multilevel analysis
Source: Cancer Med. 2021 Aug 19;10(18):6468–79. doi: 10.1002/cam4.4175 (PMC8446571; doi:10.1002/cam4.4175)
Supplement: Supplementary file 3 — Table S3 [file CAM4-10-6468-s002.docx]

| Supplement Table 3: Logistic Regression Models for Late-stage GCA Diagnosis by Location including Unknown Stage, Adults 18-89, 2004-2016 | | | | | | | | |
| --- | --- | --- | --- | --- | --- | --- | --- | --- |
|  | **SEER** | | **Texas** | | **Texas w/o STX** | | **STX** | |
| **n** | 71,552 | | 12,807 | | 10,053 | | 2,754 | |
|  | **OR** | **p-value** | **OR** | **p-value** | **OR** | **p-value** | **OR** | **p-value** |
| **Sex** |  |  |  |  |  |  |  |  |
| Female | Ref |  | Ref |  | Ref |  | Ref |  |
| Male | **1.052** | **0.0025** | 1.037 | 0.3520 | 1.049 | 0.2812 | 1.002 | 0.9860 |
| **Age at DX** |  |  |  |  |  |  |  |  |
| 20-39 | **2.661** | **<0.0001** | **2.815** | **<0.0001** | **2.569** | **<0.0001** | **4.018** | **<0.0001** |
| 40-64 | **1.659** | **<0.0001** | **1.783** | **<0.0001** | **1.694** | **<0.0001** | **2.171** | **<0.0001** |
| 65+ | Ref |  | Ref |  | Ref |  | Ref |  |
| **Race/Ethnicity** |  |  |  |  |  |  |  |  |
| NH White | Ref |  | Ref |  | Ref |  | Ref |  |
| NH Black | 1.011 | 0.6653 | 1.013 | 0.8289 | 1.036 | 0.5803 | 0.779 | 0.3478 |
| Latino | 1.025 | 0.3005 | 1.095 | 0.0676 | **1.128** | **0.0284** | 1.089 | 0.4641 |
| NH Others | **0.673** | **<0.0001** | **0.815** | **0.0241** | **0.820** | **0.0332** | 1.018 | 0.9649 |
| **Anatomical Site** |  |  |  |  |  |  |  |  |
| Cardia | Ref |  | Ref |  | Ref |  | Ref |  |
| Non-Cardia | **0.893** | **<0.0001** | 0.923 | 0.1103 | 0.902 | 0.0622 | 1.026 | 0.8271 |
| Overlap | **1.499** | **<0.0001** | **1.373** | **<0.0001** | **1.451** | **<0.0001** | 1.239 | 0.2175 |
| NOS | **1.728** | **<0.0001** | **1.342** | **<0.0001** | **1.292** | **<0.0001** | **1.535** | **0.0013** |
| **Year of DX** |  |  |  |  |  |  |  |  |
| 2004-2007 | Ref |  | Ref |  | Ref |  | Ref |  |
| 2008-2011 | 1.036 | 0.0842 | 1.017 | 0.7256 | 0.994 | 0.9152 | 1.147 | 0.2015 |
| 2012-2016 | **1.154** | **<0.0001** | **1.149** | **0.0022** | **1.116** | **0.0299** | **1.325** | **0.0064** |
| **County Level Indicators** |  |  |  |  |  |  |  |  |
| % Smokers (z-score) | **0.923** | **<0.0001** | 0.997 | 0.9691 | 0.901 | 0.1050 | 1.267 | 0.0942 |
| % Obese (z-score) | 1.034 | 0.0865 | 0.961 | 0.4305 | 0.994 | 0.9026 | 0.922 | 0.5027 |
| % Excessive Alcohol (z-score) | 1.011 | 0.3652 | 1.071 | 0.0955 | 1.011 | 0.7892 | 1.123 | 0.1646 |
| Food Environment Index (z-score) | **0.966** | **0.0236** | 0.988 | 0.7144 | 0.983 | 0.6002 | 1.090 | 0.3211 |
| **Social Deprivation Index** |  |  |  |  |  |  |  |  |
| SDI 0-20 (least deprived) | Ref |  | Ref |  | Ref |  | Ref |  |
| SDI 21-79 | 1.035 | 0.3080 | 1.169 | 0.0804 | **1.209** | **0.0148** | 0.985 | 0.9448 |
| SDI 80-100 (most deprived) | 0.951 | 0.2431 | 1.165 | 0.1383 | 1.153 | 0.0668 | 1.063 | 0.8319 |

Also adjusted for reporting source
