# Supplementary material for: Gastric adenocarcinoma burden and late‐stage diagnosis in Latino and non‐Latino populations in the United States and Texas, during 2004–2016: A multilevel analysis
Source: Cancer Med. 2021 Aug 19;10(18):6468–79. doi: 10.1002/cam4.4175 (PMC8446571; doi:10.1002/cam4.4175)
Supplement: Supplementary file 4 — Table S4 [file CAM4-10-6468-s007.docx]

| Supplement Table 4: Logistic Regression Models for Late-stage GCA Diagnosis by Anatomic Site including Unknown Stage, Adults 18-89, 2004-2016 | | | | | | | | |
| --- | --- | --- | --- | --- | --- | --- | --- | --- |
|  | **Cardia** | | **Non-Cardia** | | **Overlap** | | **NOS** | |
| **n** | 26,170 | | 37,971 | | 6,106 | | 14,112 | |
|  | **OR** | **p-value** | **OR** | **p-value** | **OR** | **p-value** | **OR** | **p-value** |
| **Location** |  |  |  |  |  |  |  |  |
| SEER | Ref |  | Ref |  | Ref |  | Ref |  |
| TX (w/o) STX | **0.909** | **0.0477** | **0.874** | **0.0080** | 0.892 | 0.2413 | **0.640** | **<0.0001** |
| STX | **0.659** | **<0.0001** | **0.814** | **0.0098** | **0.541** | **<0.0001** | **0.584** | **<0.0001** |
| **Sex** |  |  |  |  |  |  |  |  |
| Female | Ref |  | Ref |  | Ref |  | Ref |  |
| Male | 1.031 | 0.3296 | **1.100** | **<0.0001** | 1.044 | 0.4189 | 0.987 | 0.7112 |
| **Age at DX** |  |  |  |  |  |  |  |  |
| 20-39 | **2.366** | **<0.0001** | **2.911** | **<0.0001** | **2.561** | **<0.0001** | **2.770** | **<0.0001** |
| 40-64 | **1.489** | **<0.0001** | **1.717** | **<0.0001** | **1.716** | **<0.0001** | **2.019** | **<0.0001** |
| 65+ | Ref |  | Ref |  | Ref |  | Ref |  |
| **Race/Ethnicity** |  |  |  |  |  |  |  |  |
| NH White | Ref |  | Ref |  | Ref |  | Ref |  |
| NH Black | **1.256** | **<0.0001** | 0.945 | 0.0927 | 0.989 | 0.8871 | 0.916 | 0.1033 |
| Latino | **1.122** | **0.0093** | 0.987 | 0.6737 | 1.138 | 0.0682 | 0.952 | 0.3215 |
| NH Others | **0.837** | **0.0020** | **0.637** | **<0.0001** | **0.848** | **0.0459** | **0.600** | **<0.0001** |
| **Year of DX** |  |  |  |  |  |  |  |  |
| 2004-2007 | Ref |  | Ref |  | Ref |  | Ref |  |
| 2008-2011 | **1.070** | **0.0473** | 1.011 | 0.6967 | 1.019 | 0.7744 | 1.030 | 0.5024 |
| 2012-2016 | **1.217** | **<0.0001** | **1.074** | **0.0076** | 1.067 | 0.3082 | **1.293** | **<0.0001** |
| **County Level Indicators** |  |  |  |  |  |  |  |  |
| % Smokers (z-score) | **0.938** | **0.0061** | **0.948** | **0.0371** | **0.892** | **0.0302** | **0.865** | **0.0004** |
| % Obese (z-score) | 1.038 | 0.1174 | 1.016 | 0.5490 | 1.082 | 0.1271 | 1.019 | 0.6431 |
| % Excessive Alcohol (z-score) | 1.004 | 0.7940 | **1.035** | **0.0288** | 1.042 | 0.1717 | 0.962 | 0.1308 |
| Food Environment Index (z-score) | 1.002 | 0.9280 | **0.958** | **0.0274** | 0.987 | 0.7445 | 0.950 | 0.0768 |
| **Social Deprivation Index** |  |  |  |  |  |  |  |  |
| SDI 0-20 (least deprived) | Ref |  | Ref |  | Ref |  | Ref |  |
| SDI 21-79 | 1.022 | 0.5684 | 1.051 | 0.2738 | 1.048 | 0.5956 | **1.160** | **0.0387** |
| SDI 80-100 (most deprived) | 0.972 | 0.5311 | 1.007 | 0.8940 | 1.013 | 0.8926 | 1.001 | 0.9864 |

Also adjusted for reporting source.
