# Supplementary material for: Gastric adenocarcinoma burden and late‐stage diagnosis in Latino and non‐Latino populations in the United States and Texas, during 2004–2016: A multilevel analysis
Source: Cancer Med. 2021 Aug 19;10(18):6468–79. doi: 10.1002/cam4.4175 (PMC8446571; doi:10.1002/cam4.4175)
Supplement: Supplementary file 5 — Table S5 [file CAM4-10-6468-s003.docx]

| Supplemental Table 5: Descriptive Statistics and Multilevel Logistic Regression Models for Late-stage GCA Diagnosis, Adults 20+, 2011-2015 (SDI timeframe) | | | | | | | | | |
| --- | --- | --- | --- | --- | --- | --- | --- | --- | --- |
|  | **Descriptive Statistics** | | | **Logistic Regression Models** | | | | | |
|  | **All GCA Patients** | **Patients with Late-Stage GCA DX** | | **Model 1** | | **Model 2** | | **Model 3** | |
| **n** | 29,915 | 13,447 | | 29,915 | | 29,915 | | 29,915 | |
|  | **% (n)** | **% (n)** | **p-value** | **OR** | **p-value** | **OR** | **p-value** | **OR** | **p-value** |
| **Late-Stage GCA DX** |  |  | --- |  |  |  |  |  |  |
| Yes | 44.95% (13,447) | --- |  | --- | --- | --- | --- | --- | --- |
| No | 55.05% (16,468) | --- |  | --- | --- | --- | --- | --- | --- |
| **Location** |  |  | 0.0014 |  |  |  |  |  |  |
| SEER | 85.40% (25,546) | 44.61% (11,397) |  | Ref |  | Ref |  | Ref |  |
| TX (w/o) STX | 11.53% (3,450) | 47.80% (1,649) |  | 1.068 | 0.1690 | 0.977 | 0.6257 | 0.954 | 0.3592 |
| STX | 3.07% (919) | 43.63% (401) |  | 0.962 | 0.6460 | 0.860 | 0.0894 | 0.865 | 0.0983 |
| **Sex** |  |  | 0.8991 |  |  |  |  |  |  |
| Female | 35.37% (10,580) | 45.00% (4,761) |  |  |  | Ref |  | Ref |  |
| Male | 64.63% (19,335) | 44.92% (8,686) |  | --- | --- | 1.000 | 0.9937 | 0.999 | 0.9743 |
| **Age at DX** |  |  | <0.0001 |  |  |  |  |  |  |
| 20-39 | 3.86% (1,154) | 63.43% (732) |  | --- | --- | **2.522** | **<0.0001** | **2.526** | **<0.0001** |
| 40-64 | 41.63% (12,455) | 50.08% (6,238) |  | --- | --- | **1.511** | **<0.0001** | **1.512** | **<0.0001** |
| 65+ | 54.51% (16,306) | 39.72% (6,477) |  |  |  | Ref |  | Ref |  |
| **Race/Ethnicity** |  |  | <0.0001 |  |  |  |  |  |  |
| NH White | 50.39% (15,073) | 45.14% (6,804) |  |  |  | Ref |  | Ref |  |
| NH Black | 12.96% (3,877) | 46.01% (1,784) |  | --- | --- | 0.988 | 0.7513 | 0.979 | 0.6050 |
| Latino | 22.52% (6,736) | 49.04% (3,303) |  | --- | --- | 1.052 | 0.1417 | 1.047 | 0.1903 |
| NH Others | 14.14% (4,229) | 36.79% (1,556) |  | --- | --- | **0.686** | **<0.0001** | **0.678** | **<0.0001** |
| **Anatomical Site** |  |  | <0.0001 |  |  |  |  |  |  |
| Cardia | 33.74% (10,094 | 44.14% (4,455) |  |  |  | Ref |  | Ref |  |
| Non-Cardia | 44.60% (13,341) | 38.54% (5,142) |  | --- | --- | **0.843** | **<0.0001** | **0.843** | **<0.0001** |
| Overlap | 7.58% (2,268) | 52.03% (1,180) |  | --- | --- | **1.404** | **<0.0001** | **1.404** | **<0.0001** |
| NOS | 14.08% (4,212) | 63.39% (2,670) |  | --- | --- | **2.299** | **<0.0001** | **2.296** | **<0.0001** |
| **County Level Indicators Mean (Std)** |  |  |  |  |  |  |  |  |  |
| % Smokers | 14.116 (3.410) | 14.145 (3.427) | 0.1930 | --- | --- | --- | --- | 0.983 | 0.4950 |
| % Obese | 26.112 (4.921) | 26.269 (4.903) | 0.0677 | --- | --- | --- | --- | 0.976 | 0.3611 |
| % Excessive Alcohol | 18.133 (2.344) | 18.412 (2.350) | 0.2541 | --- | --- | --- | --- | 0.992 | 0.6050 |
| Food Environment Index | 7.934 (0.862) | 7.913 (0.859) | 0.0001 | --- | --- | --- | --- | **0.961** | **0.0449** |
| **Social Deprivation Index** |  |  | 0.0610 |  |  |  |  |  |  |
| SDI 0-20 (least deprived) | 14.27% (4,270) | 43.37% (1,852) |  |  |  |  |  | Ref |  |
| SDI 21-79 | 49.54% (14,821) | 45.42% (6,731) |  | --- | --- | --- | --- | 1.063 | 0.1713 |
| SDI 80-100 (most deprived) | 36.18% (10,824) | 44.94% (4,864) |  | --- | --- | --- | --- | 1.002 | 0.9667 |

Adjusted for Reporting Source. GCA: Gastric Adenocarcinoma; DX: Diagnosis
