# Supplementary material for: Gastric adenocarcinoma burden and late‐stage diagnosis in Latino and non‐Latino populations in the United States and Texas, during 2004–2016: A multilevel analysis
Source: Cancer Med. 2021 Aug 19;10(18):6468–79. doi: 10.1002/cam4.4175 (PMC8446571; doi:10.1002/cam4.4175)
Supplement: Supplementary file 6 — Table S6 [file CAM4-10-6468-s004.docx]

| Supplement Table 6: Logistic Regression Models for Late-stage GCA Diagnosis by Location including Unknown Stage, Adults 18-89, 2011-2015 (SDI timeframe) | | | | | | | | |
| --- | --- | --- | --- | --- | --- | --- | --- | --- |
|  | **SEER** | | **Texas** | | **Texas w/o STX** | | **STX** | |
| **n** | 25,546 | | 4,369 | | 3,450 | | 919 | |
|  | **OR** | **p-value** | **OR** | **p-value** | **OR** | **p-value** | **OR** | **p-value** |
| **Sex** |  |  |  |  |  |  |  |  |
| Female | Ref |  | Ref |  | Ref |  | Ref |  |
| Male | 1.005 | 0.8708 | 0.971 | 0.6611 | 0.956 | 0.5540 | 1.034 | 0.8160 |
| **Age at DX** |  |  |  |  |  |  |  |  |
| 20-39 | **2.500** | **<0.0001** | **2.702** | **<0.0001** | **2.574** | **<0.0001** | **3.091** | **0.0056** |
| 40-64 | **1.505** | **<0.0001** | **1.563** | **<0.0001** | **1.492** | **<0.0001** | **1.806** | **<0.0001** |
| 65+ | Ref |  | Ref |  | Ref |  | Ref |  |
| **Race/Ethnicity** |  |  |  |  |  |  |  |  |
| NH White | Ref |  | Ref |  | Ref |  | Ref |  |
| NH Black | 0.995 | 0.9051 | 0.910 | 0.3749 | 0.938 | 0.5620 | 0.775 | 0.5656 |
| Latino | 1.018 | 0.6412 | 1.141 | 0.1199 | 1.193 | 0.0589 | 1.020 | 0.9167 |
| NH Others | **0.664** | **<0.0001** | 0.830 | 0.2043 | 0.855 | 0.3024 | 0.662 | 0.5583 |
| **Anatomical Site** |  |  |  |  |  |  |  |  |
| Cardia | Ref |  | Ref |  | Ref |  | Ref |  |
| Non-Cardia | **0.839** | **<0.0001** | 0.860 | 0.0665 | 0.838 | 0.0507 | 0.947 | 0.7808 |
| Overlap | **1.436** | **<0.0001** | 1.208 | 0.1515 | 1.204 | 0.2227 | 1.272 | 0.3844 |
| NOS | **2.342** | **<0.0001** | **2.096** | **<0.0001** | **2.039** | **<0.0001** | **2.291** | **0.0004** |
| **County Level Indicators** |  |  |  |  |  |  |  |  |
| % Smokers (z-score) | 0.967 | 0.2162 | 1.097 | 0.4172 | 0.910 | 0.4667 | **1.753** | **0.0231** |
| % Obese (z-score) | 0.985 | 0.5788 | 0.903 | 0.2079 | 0.934 | 0.4444 | 0.994 | 0.9780 |
| % Excessive Alcohol (z-score) | 0.995 | 0.7737 | 1.052 | 0.4456 | 1.023 | 0.7991 | 1.185 | 0.2230 |
| Food Environment Index (z-score) | **0.948** | **0.0138** | 0.957 | 0.4178 | 0.949 | 0.3813 | 0.871 | 0.3755 |
| **Social Deprivation Index** |  |  |  |  |  |  |  |  |
| SDI 0-20 (least deprived) | Ref |  | Ref |  | Ref |  | Ref |  |
| SDI 21-79 | 1.071 | 0.1495 | 1.024 | 0.8637 | 1.154 | 0.3437 | 0.891 | 0.7832 |
| SDI 80-100 (most deprived) | 0.971 | 0.6056 | 1.156 | 0.3614 | 1.164 | 0.3023 | 0.928 | 0.8895 |

Also adjusted for reporting source
