# Supplementary material for: Gastric adenocarcinoma burden and late‐stage diagnosis in Latino and non‐Latino populations in the United States and Texas, during 2004–2016: A multilevel analysis
Source: Cancer Med. 2021 Aug 19;10(18):6468–79. doi: 10.1002/cam4.4175 (PMC8446571; doi:10.1002/cam4.4175)
Supplement: Supplementary file 7 — Table S7 [file CAM4-10-6468-s005.docx]

| Supplement Table 7: Logistic Regression Models for Late-stage GCA Diagnosis by Anatomic Site including Unknown Stage, Adults 18-89, 2011-2015 (SDI timeframe) | | | | | | | | |
| --- | --- | --- | --- | --- | --- | --- | --- | --- |
|  | **Cardia** | | **Non-Cardia** | | **Overlap** | | **NOS** | |
| **n** | 10,094 | | 13,341 | | 2,268 | | 4,212 | |
|  | **OR** | **p-value** | **OR** | **p-value** | **OR** | **p-value** | **OR** | **p-value** |
| **Location** |  |  |  |  |  |  |  |  |
| SEER | Ref |  | Ref |  | Ref |  | Ref |  |
| TX (w/o) STX | 0.964 | 0.6194 | 0.983 | 0.8234 | 0.899 | 0.5449 | 1.000 | 0.9999 |
| STX | 0.764 | 0.0858 | 0.965 | 0.7770 | 0.662 | 0.1139 | 0.847 | 0.3343 |
| **Sex** |  |  |  |  |  |  |  |  |
| Female | Ref |  | Ref |  | Ref |  | Ref |  |
| Male | 0.961 | 0.4285 | 1.049 | 0.1916 | 1.031 | 0.7283 | 0.920 | 0.2026 |
| **Age at DX** |  |  |  |  |  |  |  |  |
| 20-39 | **3.125** | **<0.0001** | **2.443** | **<0.0001** | **2.438** | **<0.0001** | **2.406** | **<0.0001** |
| 40-64 | **1.374** | **<0.0001** | **1.598** | **<0.0001** | **1.505** | **<0.0001** | **1.703** | **<0.0001** |
| 65+ | Ref |  | Ref |  | Ref |  | Ref |  |
| **Race/Ethnicity** |  |  |  |  |  |  |  |  |
| NH White | Ref |  | Ref |  | Ref |  | Ref |  |
| NH Black | **1.314** | **0.0026** | 0.898 | 0.0553 | 0.953 | 0.7225 | 0.871 | 0.1611 |
| Latino | 1.049 | 0.4900 | 1.033 | 0.5276 | 1.108 | 0.3811 | 0.912 | 0.3019 |
| NH Others | **0.775** | **0.0041** | **0.624** | **<0.0001** | 0.832 | 0.1788 | **0.616** | **<0.0001** |
| **County Level Indicators** |  |  |  |  |  |  |  |  |
| % Smokers (z-score) | 0.936 | 0.0643 | 1.024 | 0.5665 | 0.917 | 0.3434 | 0.998 | 0.9777 |
| % Obese (z-score) | 1.028 | 0.4510 | 0.928 | 0.0746 | 1.167 | 0.0935 | 0.909 | 0.1259 |
| % Excessive Alcohol (z-score) | 0.999 | 0.9639 | 0.997 | 0.8925 | 1.085 | 0.1332 | **0.901** | **0.0050** |
| Food Environment Index (z-score) | 0.964 | 0.2231 | **0.937** | **0.0360** | 1.037 | 0.5983 | 0.984 | 0.7154 |
| **Social Deprivation Index** |  |  |  |  |  |  |  |  |
| SDI 0-20 (least deprived) | Ref |  | Ref |  | Ref |  | Ref |  |
| SDI 21-79 | 1.058 | 0.3267 | 1.083 | 0.2786 | 1.171 | 0.3162 | 1.164 | 0.1550 |
| SDI 80-100 (most deprived) | 1.027 | 0.7028 | 1.026 | 0.7633 | 1.020 | 0.9117 | 1.091 | 0.4395 |

Also adjusted for reporting source.
